# A Review of Transcranial Magnetic Stimulation and Multimodal Neuroimaging to Characterize Post-Stroke Neuroplasticity

**DOI:** 10.3389/fneur.2015.00226

**Published:** 2015-10-29

**Authors:** Angela M. Auriat, Jason L. Neva, Sue Peters, Jennifer K. Ferris, Lara A. Boyd

**Affiliations:** ^1^Department of Physical Therapy, Faculty of Medicine, University of British Columbia, Vancouver, BC, Canada; ^2^Graduate Program in Neuroscience, Faculty of Medicine, University of British Columbia, Vancouver, BC, Canada

**Keywords:** multimodal neuroimaging, stroke, sensorimotor recovery, diffusion tensor imaging, magnetic resonance spectroscopy, functional MRI, electroencephalography, transcranial magnetic stimulation

## Abstract

Following stroke, the brain undergoes various stages of recovery where the central nervous system can reorganize neural circuitry (neuroplasticity) both spontaneously and with the aid of behavioral rehabilitation and non-invasive brain stimulation. Multiple neuroimaging techniques can characterize common structural and functional stroke-related deficits, and importantly, help predict recovery of function. Diffusion tensor imaging (DTI) typically reveals increased overall diffusivity throughout the brain following stroke, and is capable of indexing the extent of white matter damage. Magnetic resonance spectroscopy (MRS) provides an index of metabolic changes in surviving neural tissue after stroke, serving as a marker of brain function. The neural correlates of altered brain activity after stroke have been demonstrated by abnormal activation of sensorimotor cortices during task performance, and at rest, using functional magnetic resonance imaging (fMRI). Electroencephalography (EEG) has been used to characterize motor dysfunction in terms of increased cortical amplitude in the sensorimotor regions when performing upper limb movement, indicating abnormally increased cognitive effort and planning in individuals with stroke. Transcranial magnetic stimulation (TMS) work reveals changes in ipsilesional and contralesional cortical excitability in the sensorimotor cortices. The severity of motor deficits indexed using TMS has been linked to the magnitude of activity imbalance between the sensorimotor cortices. In this paper, we will provide a narrative review of data from studies utilizing DTI, MRS, fMRI, EEG, and brain stimulation techniques focusing on TMS and its combination with uni- and multimodal neuroimaging methods to assess recovery after stroke. Approaches that delineate the best measures with which to predict or positively alter outcomes will be highlighted.

## Introduction

Recent advances in stroke treatment have stressed early intervention, greatly reducing the risk of mortality after stroke (1). Yet, development of treatments aimed at improving function after stroke has failed to keep pace, in part because rehabilitation specialists do not yet understand how to best help the brain recover from stroke. The importance of this issue is underscored by work from the Boyd Lab showing a *clinically meaningful decline* in population-based quality of life for Canadians with stroke from 1998 to 2005 (2). In this work, declines in health-related quality of life in the Canadian population were associated with increases in the proportion of individuals with impaired motor function post-stroke. Together the high incidence, increased survival rates, and decreased quality of life following stroke demonstrate a critical need for improved understanding of brain recovery after stroke.

**Figure 1:**
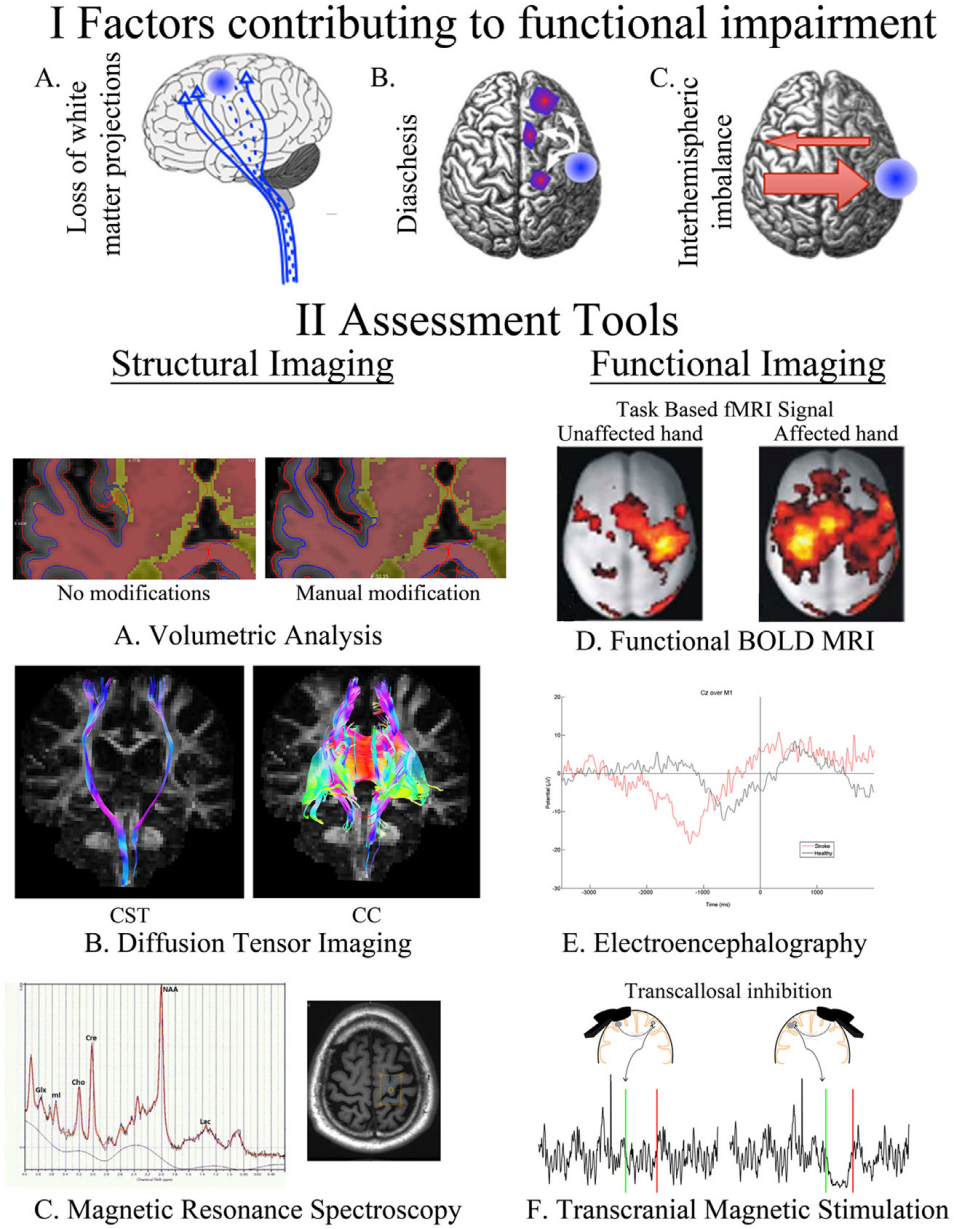
**A summary of our current understanding of factors that contribute to post-stroke impairment (I) and the assessment tools available for quantifying these changes (II)**. Loss of white matter projections is illustrated as a decreased number of CST projections (IA). Diaschisis, a remote functional depression, can impact intra- and interhemispheric areas (IB). A focal lesion can disrupt the mutual balanced inhibition between hemispheres. Damage from stroke disrupts the balance by decreasing the inhibition of the contralesional hemisphere, which results in increased inhibition of the injured hemisphere (IC). FreeSurfer-based volumetric analysis, using structural T1s, can be manually modified to correct for errors in the automated segmentation of injured brains (IIA). Identification of CST and CC in an individual with chronic stroke utilizing tractography of diffusion-weighted images (IIB). The axial brain image identifies the voxel placement in the hand knob of an individual with chronic stroke, the resulting spectra quantifies multiple neurotransmitters (IIC). BOLD signal during movements of the unaffected and affected hand in individuals with left-sided subcortical stroke; modified, with permission, from Grefkes et al. (16) (IID). EEG trace from an electrode located at Cz (over primary motor cortex) in an individual with chronic stroke and a healthy control as they take a step (time 0) (IIE). Transcallosal inhibition evoked from stimulation over ipsilesional and contralesional primary motor cortex (IIF). Ipsilesional stimulation failed to produce an observable iSP in the ipsilateral (to the TMS pulse) limb, whereas contralesional stimulation evoked a quantifiable iSP in the ipsilateral (to the TMS pulse) limb. iSP occurs in the time between the green (onset) and red (offset) lines. CST, cortical spinal tract; CC, corpus callosum; fMRI, functional MRI; BOLD, blood oxygen level dependent; iSP, ipsilateral silent period; TMS, transcranial magnetic stimulation.

Many have attempted to define the neural mechanisms of post-stroke impairment and recovery in the hope that understanding these processes will improve rehabilitation interventions and enhance function (Figure 1, Part I). Since the development of neuroimaging techniques, such as magnetic resonance imaging (MRI) and functional MRI (fMRI), it is possible to identify both structural and functional brain changes, termed neuroplasticity, as individuals with stroke re-learn motor skills. In addition, the use of transcranial magnetic stimulation (TMS) allows cortical excitability to be temporarily enhanced or reduced, which enables researchers to experimentally test the influence of specific brain regions on motor learning and recovery from stroke. To date, numerous studies show neuroplastic change after stroke by documenting recovery of function that is independent of spontaneous change associated with acute recovery (3, 4). Our work (3, 5–9) and that of others (10–12) clearly shows that motor learning and capacity for neuroplastic change (13, 14) are preserved, even during the chronic stage after stroke. Experience-dependent neuroplasticity likely explains a portion of the change associated with motor learning after stroke in this work (15), yet despite these advances in knowledge, no clear pattern of motor-related brain activation has emerged that fully explains how the brain compensates for stroke-related damage during motor learning.

In part, our failure to grasp how the damaged brain learns stems from an incomplete understanding of the relationships between behavior and brain function. Key to improving functional recovery after stroke is more fully understanding and mapping experience-dependent neuroplasticity (17), which demonstrates that the functional organization of the motor system can be modified by use. Technological advances have enabled detailed structural assessment of the brain with volumetric analysis of white and gray matter, the indexing of white matter connectivity using diffusion imaging, quantifying metabolic changes with magnetic resonance spectroscopy (MRS), mapping of brain activity with fMRI and electroencephalography (EEG), and assessing experience-dependent neuroplasticity through the manipulation of cortical excitability using repetitive TMS (rTMS). In this review, we highlight the use of these neuroimaging techniques to map the neuroplasticity of motor learning and sensorimotor recovery, as well as the advances in knowledge that have been stimulated from their use. In combination, the knowledge gained from these approaches is contributing significantly to the genesis of novel, evidence-based interventions designed to promote functional recovery after stroke.

## Neuroimaging

### Structural Imaging

#### Volumetric Analysis

It has long been recognized that lesion location rather than size explains the bulk of neurological deficits after stroke (18). For instance, the degree damage to the cortical spinal tract (CST) rather than lesion volume correlates with motor ability after stroke (19). However, stroke-related damage also has effects on regions remote from the site of injury (20). The time point of assessment is important because of delayed atrophy in areas remote from the stroke (21). Advances in volumetric analysis of MRI have allowed for the automated quantification of brain volumes after segmentation into gray and white matter (22, 23) often using only an anatomical T1 scan (Figure 1, Section IIA). The quality of the scan influences the precision of segmentation and having additional scans, such as fluid-attenuated inversion recovery (FLAIR), T2, or proton density (PD), can improve accuracy and identification of subtle lesions (23). Unfortunately, difficulties arise when using these methods to quantify brains with a neurological pathology (24, 25). Caution must be taken to ensure programs designed to use anatomical landmarks to segment and quantify brain volumes are functioning as expected with analysis of chronic post-stroke brains, where landmarks may shift or be non-existent due to direct damage or atrophy. Recently, our group has utilized the FreeSurfer-based image analysis package (26, 27) for volumetric segmentation in chronic stroke and found that segmentation was unaffected by small subcortical lesions (24). However, participants with more extensive damage had to be excluded from the analysis due to segmentation errors. Alternative segmentation programs or using more extensive manual edits will allow for the inclusion of participants with larger lesions.

Volumetric analysis may be a valuable predictor of responders to post-stroke interventions (23, 28). Future use of volumetric analysis in rehabilitation studies will likely provide more useful information on the influence of structural integrity on post-stroke recovery. However, extreme caution and manual review/intervention of computerized assessments must be used to ensure accurate quantification of post-stroke brains (24, 25).

#### Diffusion-Weighted Imaging

Diffusion-weighted magnetic resonance imaging (DW-MRI) non-invasively provides information on white matter pathways in the human brain. Based on its ability to determine water diffusion characteristics, DW-MRI has been extensively used to identify the orientation and integrity of white matter after stroke, and to relate these measures to motor function [see Ref. (28) for review]. Brain regions, such as the corpus callosum (CC) (29, 30) and the corticospinal tract (CST) (29, 31–33), have been repeatedly studied and related to both motor function and functional potential (30, 31, 33). DW-MRI has been touted as a promising tool for rehabilitation planning and prognosis after stroke (31), and may predict neural changes after motor learning. Importantly, preliminary studies have demonstrated that the integrity of CST (24, 34) and CC (35) influences the efficacy of rTMS, suggesting that DW-MRI can provide valuable information when selecting rTMS protocols and predicting the efficacy of an intervention.

The motion of water molecules is restricted based on its location and in white matter movement of water is restricted across the tracts, with a relatively greater freedom of movement parallel to the white matter fibers. It is this basic principle, which allows for DW-MRI to identify the diffusion characteristics of white matter and predict specific white matter pathways. Several diffusion-based measures have been related to post-stroke outcome, primarily, fractional anisotropy (FA), apparent diffusion coefficient (ADC), axial diffusivity (AD), radial diffusivity (RD), number of tracts, and tract volume. FA is the most commonly reported DW-MRI measure, and indicates the degree of directionality within the tissue microstructure, which is determined by tissue features, such as axons, myelin, and microtubules. FA ranges from 0 (completely isotropic) to 1 (completely anisotropic); therefore, higher FA indicates greater directionality (36, 37). ADC, AD, and RD are all based on the eigenvalues of the apparent diffusion tensor [λ_1_, λ_2_, and λ_3_ (38)]. AD is an indicator of water diffusion along the parallel, principal, direction of axonal water diffusion [AD = λ_1_ (38)]. RD is an index of water diffusion perpendicular to the principal direction of water [RD = λ_2_ + λ_3_/2 (38)]. ADC is the mean value of eigenvalues of the apparent diffusion tensor [ADC = λ_1_ + λ_2_ + λ_3_/3 (38)]. Tractography methods allow for the visualization of fiber architecture and also allow for the identification of fiber number and volume in pathways of interest to stroke recovery (Figure 1, Section IIB).

Although the reproducibility of tractography has been established in a stroke population (39, 40), different analysis methods can affect the interpretation of results (41). At present, no “gold standard” method for fiber tractography exists for *in vivo* application (42–44). For example, our group has recently found that diffusion tensor imaging (DTI) and constrained spherical deconvolution (CSD) methods produce significantly different results when applied to individuals with chronic stroke (41). Although DTI is the most commonly applied method of tractography analysis in stroke research, CSD analysis provided a stronger relationship between CST and CC white matter characteristics, and post-stroke outcome. Additionally, DTI-based tractography often fails to reconstruct fibers projecting to the lateral aspect of the cortex (41, 42). Lateral projections of the CST play a significant role in motor recovery after stroke (45), specifically fine motor control of the hand (46). The failure of DTI to detect these lateral projections likely hinders correlations between CST and CC diffusion measures and motor function. If DW-MRI tractography is to become a feasible tool for assessing prognosis, functional potential, or rehabilitation strategies, it is important that this technique be as sensitive and specific to actual white matter fiber architecture as possible. Inability to detect an intact CST or an under-estimation of the projection of fiber populations may undermine patients’ expected potential for recovery resulting in minimized rehabilitation efforts. Additional studies are needed to identify optimized tractography strategies for identifying the fiber projections important for stroke recovery.

In addition to tractography, several strategies are utilized to interpret the microstructural white matter information provided from DW-MRI. Many studies use a FA map to place a region of interest (ROI) over a section of white matter (29), or use tract-based spatial statistics (TBSS) to isolate specific regions of change (47). Each of these methods has been able to correlate FA and/or diffusion measures of the CST with sensorimotor function and impairment following stroke (29, 32, 48). Lindenberg et al. found a correlation between fiber number asymmetry (ipsilesional − contralesional/ipsilesional + contralesional) and motor outcome in chronic stroke (32). Cho et al. used DTI tractography to classify CST integrity after corona radiata infarct (49) and intra-cerebral hemorrhage (50), and found a relationship between tract involvement and functional outcome. ADC of the CST appears to be elevated in the chronic stage of stroke (51, 52), and has been related to functional outcomes (28, 52). AD and RD have been less frequently reported after stroke. Nonetheless, studies in individuals with acute stroke found AD of the CST to be related to motor outcomes (53, 54). One study found increased RD in several regions, including the posterior CC, in acute stroke patients compared to controls; however, increased AD occurred only in the corona radiata (55). These results are consistent with the work by Lindenberg et al., who assessed individuals with chronic stroke in comparison to controls (30). Recent work has shown that ADC, AD, and RD are elevated in the ipsilesional CST and are related to motor outcome in individuals with chronic stroke (41).

Several studies have assessed the relationship between DW-MRI-based diffusion measures of the CC and post-stroke outcome. Recently, Takenobu et al. used a combination of voxel-based statistical tractography and a deterministic ROI-based approach to determine callosal FA in acute ischemic stroke patients (47). A significant positive correlation between FA values within a ROI placed in the callosal midbody and motor impairment was reported. Lindenberg and colleagues employed a probabilistic tractography method, identifying white matter tracts passing through contralesional primary motor cortex, and found that several DTI-based outcomes were related to baseline motor function and improvements in motor function after a 5-day intervention combining non-invasive brain stimulation and motor practice (30). Specifically, transcallosal FA was negatively correlated with baseline motor function, and both AD and RD were positively correlated with change in function between pre- and post-intervention assessments.

Together these findings indicate multiple measures of white matter microstructure of the CST and CC correlate with stroke outcome. It remains to be seen which diffusion measure(s) and method(s) will provide the most reliable indication of CST and CC function. Populations with stroke tend to have heterogeneous characteristics, such as, varied time since stroke onset, wide range of functional and cognitive impairments, and differences in lesion size and location. The contribution of these factors to white matter microstructure have not been comprehensively explored, and should be evaluated in future work to enhance the use of DW-MRI to predict stroke outcome and the response to interventions.

#### Magnetic Resonance Spectroscopy

Magnetic resonance spectroscopy allows for the non-invasive measurement of metabolites *in vivo*, within a defined region of tissue. H^1^MRS uses resonance signals from hydrogen protons to quantify cerebral metabolites, which have different identifiable resonance signals (or peaks) in a static magnetic field, measured in parts per million (ppm). Thus, the magnitude of the peak resonance at the chemical shift point for each metabolite can be measured and a spectral map computed, providing information on the presence and concentration of metabolites within the target tissue [see Ref. (56) for review] (Figure 1, Section IIC). The process of acquiring MRS data (shimming, water suppression, and phasing curve fitting) has now been automated and is available in programs, such as linear combination (LC) model (57) or magnetic resonance user interface (MRUI) (58).

The number of metabolites that can be differentiated in the MRS spectrum depends on the field strength of the MRI scanner (59, 60). With a 3T MRI, it is normally possible to obtain reliable peaks for six different metabolites: *N*-acetylaspartate (NAA), myo-inositol (mI), choline, creatine, glutamate, and lactate. Signals from the different peaks overlap making detection of less-abundant metabolites, such as gamma-aminobutyric acid (GABA), difficult without specific optimization of the MRS procedure that involves editing the spectra to obtain the GABA peak at the cost of losing information from other observable peaks (61). The physiological roles of the five identifiable metabolites are still under examination. The physiological role of NAA in the CNS is unclear; however, it is considered a marker of viable neurons. Lowered levels of NAA may indicate neural loss or death (62). mI is a cerebral osmolyte and occurs in astrocytes. It is considered a marker of glial cells and elevated mI is often considered a sign of gliosis or cytotoxic edema (63). mI is elevated in spared neural tissue in chronic stroke (64, 65). Choline represents the sum of four choline-containing compounds in the CNS, all of which are contained in cellular membranes; choline is considered a marker of cell membrane integrity. Elevated choline levels may indicate increased cell membrane turnover or demyelination (62, 66). Creatine also represents the sum of creatine-containing compounds, creatine and phosphocreatine, both of which are cellular energy reserves and are markers of energy metabolism in the brain (62). Creatine and choline levels are commonly believed to be stable across the brain and are often used to normalize levels of other cerebral metabolites; however, this may not be an appropriate approach in neuropathological conditions, such as stroke, as choline and creatine levels may be unstable after cerebral infarct (67). Glutamate represents the sum of glutamate and its precursor glutamine; it is not possible to differentiate these two compounds at 3T field strength (59). Glutamate is the principle excitatory neurotransmitter in the CNS and levels of glutamate may be of particular interest in indexing changes related to *N*-methyl-d-aspartate receptor (NMDAR)-mediated neuroplasticity, or glutamate excitotoxicity post-stroke.

Magnetic resonance spectroscopy has significant potential to act as an index of metabolic changes in surviving neural tissue after stroke (68). Thus, MRS has primarily been useful as a marker of neuronal loss or to indicate altered metabolic processes in penumbral tissue following infarction. NAA levels are reduced in areas of cerebral infarct (69), consistent with neural death, and appear to reduce further from acute to chronic stroke, perhaps indicating neuronal loss by diaschisis (70). Combining lactate peaks with NAA data provides useful predictive information about the viability of peri-infarct tissue in acute stroke (71–73). MRS has been less utilized in evaluating sensorimotor outcomes in chronic stroke, though analyses of spared ipsilesional tissue have provided interesting insights into neural adaptations in motor networks following distal infarct. In individuals with subcortical stroke, there is lower NAA and higher mI in spared ipsilesional primary motor cortex (M1) (65, 74); this is consistent with neuronal stress or atrophy as result of an infarct to the motor network. Lower NAA and higher mI have also been reported in the ipsilesional supplementary motor area (SMA) and premotor cortex, respectively (64). NAA levels in M1 and non-primary motor areas positively correlate to motor function in several reports (64, 74–76) suggesting motor outcomes after stroke rely in part on the integrity of surviving neural tissue. There have been fewer reports on neurotransmitter levels and functional outcomes after stroke. Cirstea et al. report levels of glutamate in ipsilesional M1 correlate with motor impairment, with higher levels of glutamate relating to better motor function, though glutamate was not significantly reduced in ipsilesional M1 compared to contralesional M1 (65). A recent study from Blicher et al., using optimized MRS protocols for detection of GABA, reveals GABA is reduced in ipsilesional M1 after stroke (77). Further, Blicher et al. report improvements in motor function in response to constraint-induced therapy (CIT) related to individual differences in GABA levels, with higher baseline GABA in ipsilesional M1 relating to greater improvements in motor function after CIT (77). Future studies linking MRS measures to functional outcomes are needed, particularly in relation to glutamate’s potential role in motor adaptation after stroke. MRS provides significant potential benefit as a modality to link observations of changes in neural activity post-stroke from fMRI or TMS imaging with changes in metabolic function.

Magnetic resonance spectroscopy could also be a valuable tool to advance our understanding of the neurochemical effects of rTMS, and may be used as a predictive measure to identify responders from non-responders. It is thought that rTMS does not change NAA levels, instead it shifts neuronal metabolism and neurotransmitter levels (78). Studies examining the effects of rTMS on MRS measures have, thus far, largely been conducted on high-frequency rTMS over the dorsolateral prefrontal cortex (DLPFC) in the treatment of depression. These studies have begun to examine individual variability in pre-stimulation metabolite levels and how these relate to treatment response. Participants who responded to high-frequency rTMS over DLPFC for treatment of depression showed lower baseline levels of glutamate prior to rTMS stimulation, and greater increases in cortical glutamate in response to rTMS (79, 80), while non-responders showed a decrease in glutamate levels in response to stimulation (80). Therefore, response to rTMS appears to rely in part on baseline levels of glutamate in target brain regions. These studies were conducted on rTMS for depression, with different stimulation targets and network effects than rTMS for sensorimotor recovery. However, they highlight the potential value of MRS as an index of treatment response to stimulation for stroke patients.

There is scant research to date on MRS response to rTMS over sensorimotor regions as it relates to stroke recovery. To our knowledge, only one such study has been conducted, by Stagg et al., using GABA-optimized MRS in examination of the effects of continuous theta burst stimulation (cTBS) to M1 (81). The authors report cTBS, which has inhibitory effects on cortical circuitry, increases GABA levels without affecting glutamate levels in M1 (81). Individual baselines in GABA levels relate to improvement gains on upper limb motor function (77), and a study in healthy adults demonstrated that individuals with greater reductions in GABA levels after transcranial direct-current stimulation (tDCS) showed improved motor learning and greater M1 activation in fMRI (82). It remains to be seen whether individuals with differing levels of baseline GABA following stroke show differing responses to rTMS protocols, this is an avenue that should be examined in future research. Not only would future MRS work expand our understanding of the neurobiological actions of rTMS, but it also could allow for improved understanding of baseline neurochemical characteristics that predict response to rTMS protocols, and thus more targeted individualized treatment approaches in stroke rehabilitation.

### Functional Imaging

#### Functional MRI

Functional MRI measures changes in blood movement in the brain over time. This signal is the blood oxygen level dependent (BOLD) signal. The BOLD signal is an indirect measure of neural activity and reflects the amount of deoxyhemoglobin in a tissue. The amount of deoxyhemoglobin depends on the local rate of metabolism of oxygen, the volume of blood in the region, and the amount of blood flow in a region (83). As neural activity increases in a brain region, local oxygen metabolism, blood volume, and blood flow all increase together (83). When MRI measures the BOLD signal, there is a time delay between the neural event and the signal measurement. The “fast response” occurs 2–3 s after an event with the main BOLD signal recorded ~5 s later. In the literature, the BOLD signal is sometimes described as a “hemodynamic response.”

Measurement of BOLD signal can occur as the study participant is performing a task (Figure 1, Section IID), or while “resting” – the participant is typically asked to think of nothing in particular but to remain awake (84). After collecting the study data, it can be processed and analyzed in very similar ways. The difference is that some analysis techniques are designed for use with certain experiment types (i.e., for resting state). Resting-state fMRI is defined as the spontaneous low-frequency (<0.1 Hz) BOLD fluctuations with spatio-temporal correlations in networks (85). What the BOLD signal fluctuations mean is not yet clear, but increasing evidence suggests it does have a neural basis (85).

In the past, stroke rehabilitative research using neuroimaging focused on the analysis of local lesion-specific activity and subsequent impairments (86, 87). Limitations in computation and mathematical modeling restricted study to isolated brain regions, though clinically the effects of an isolated stroke can demonstrate large sensorimotor and cognitive effects in remote areas (88). Recent advancements in technological and scientific knowledge have allowed for broader study of brain activity upstream and downstream from the stroke lesion, namely network analysis. Network analysis allows for the study of potential widespread changes in neural activity after a focal lesion. Analyzing patterns of network activity can inform researchers and clinicians of the effect a lesion has on the output of brain activity and may indicate whether certain “compensatory” network patterns are better than others for producing functional motor performance. A recent review of network analysis demonstrated altered activity both adjacent to and distant from a stroke lesion, affecting both hemispheres, and a pattern of change in network activity linked with motor impairments and recovery (89). Reorganization in the lesioned hemisphere includes interactions between the fronto-parietal regions and the primary motor cortex, which may suggest greater cortical control is needed for motor performance of the paretic upper extremity (89). These studies underline the ability of network analysis to determine connectivity patterns after a stroke, and its potential for determining the effectiveness of current rehabilitative therapies. If network analysis can link certain patterns of early post-stroke activity with better prognosis, it may have a role in informing the direction of future therapies.

Brain network activity after a stroke is commonly studied with task-based fMRI. The challenges with using fMRI in individuals after a stroke, is that the post-stroke motor impairments can make motor performance difficult often resulting in movement synergies (90), mirror movements (91), and head motion during an fMRI scan (92). If during an fMRI study, participants produce head movement beyond a few millimeters, move in synergies or produce mirror movements, the scan may be rendered useless. People who have sustained a severe stroke with resulting severe motor impairments are often not studied with task-based fMRI, as motor performance of even simple tasks are frequently not possible without assistance, though some studies attempt to overcome this limitation by studying passive movements (93, 94). Even those who have sustained a mild or moderate stroke may have difficulty performing common functional tasks, such as individuated finger movements, so researchers are limited to studying basic and simple motor tasks, limiting generalizability to other motor tasks. Imaging the brain during rest allows for the study of individuals with a wide range of post-stroke motor impairments, and permits the examination of network activity without the need for task performance. For these reasons, resting-state imaging is an attractive method for studying stroke network activity.

##### Resting-State fMRI

Resting-state fMRI can characterize functional deficits after a stroke and provide important predictive evidence that links brain behavior with functional sensorimotor recovery of the upper limb. After a cortical stroke, participants demonstrate increased network activity in the ipsilesional fronto-parietal cortex, bilateral thalamus and cerebellum, while contralesional M1 and occipital cortical activity are decreased compared with healthy controls (95). Furthermore, the functional connectivity of the ipsilesional M1 with the contralesional thalamus, SMA, and middle frontal gyrus during the acute stroke phase positively correlate with motor recovery after 6 months (95), suggesting that changes in upper extremity motor impairment can be predicted by alterations in resting-state activity. Recently, participants with impaired upper extremity function received 12 weeks of training with shoulder and elbow robotic rehabilitation (96). Resting-state fMRI and upper extremity motor impairment was assessed before and after training. Decreased impairment could be predicted from functional connectivity changes measured by resting-state fMRI. Resting-state fMRI can reveal disrupted functional connections within hours of stroke as well as during recovery. Individuals with ischemic stroke were scanned within 24 h, 1 week, and 3 months post-stroke (97). Within hours after stroke, lower connectivity was found in individuals with motor deficits. Interestingly, connectivity was restored 1 week later in those with recovered hand function. However, residual decreased subcortical connectivity remained 3 months later, even in those individuals without remaining hand motor impairment. These findings indicate that though motor function improves for some individuals after stroke, resting-state fMRI may remain altered. Resting-state fMRI also allows for the analysis of multiple networks simultaneously. Recent work has proposed that disrupted whole brain connectivity in both the sensorimotor and dorsal attention network is closely linked with functional impairment more than the intra-hemispheric connectivity (98).

##### Task-Based fMRI

Task-based fMRI can inform the capacity of individuals to recover after stroke, specifically with regard to motor function and learning. fMRI studies have found that paretic hand movement early after stroke is linked to widespread bilateral activity within the motor system, with greater bilateral activity found in individuals with greater motor impairment (99). Research directed at understanding the function of this bilateral pattern of activity suggests that the surviving brain regions influence distant regions during movement (99). It is now known that brain regions that survive the initial stroke influence one another during movement, and that multiple brain regions and pathways participate in reorganization and functional recovery, such as the CST, brainstem pathways, interhemispheric connections (100). The contralesional hemisphere also provides support for paretic hand movements (100). Task-specific practice in individuals with chronic stroke facilitated motor learning and reduced the volume of contralesional cortical activity while using the paretic arm (101). Performing the learned task altered cortical activation by producing a more normalized contralateral pattern of brain activation, which suggests task-specific motor learning may be an important stimulant for neuroplastic change and can remediate maladaptive patterns of brain activity after stroke. Our group has found motor learning and overall improvements in motor control are associated with increased response in the prefrontal-based attentional network in individuals with chronic stroke (14). Additionally, evidence of plasticity is also noted for movement of the non-paretic arm; this activity is related to alterations in neural activation in areas anatomically and functionally connected to the lesion, implying an extensive bilateral network is involved (102).

#### Electroencephalography

Electroencephalography uses surface electrodes placed on the scalp to detect fluctuating electrical voltages, which result from the small electrical currents generated by active neurons (103). EEG recordings are mainly generated by pyramidal neurons in cortical layers III, V, and VI, with summation of cortical activity producing a voltage field that can be recorded on the scalp (103). EEG is used for diagnosis, prognosis, treatment monitoring, and clinical management in acute ischemic stroke (104). Additionally, in chronic stroke, the EEG signal can identify subtle changes in the brain that cannot be detected by clinical measures; further, quantification of the EEG signal before and after rehabilitation interventions can assess neuroplasticity both locally surrounding the lesion and within whole brain networks (105).

For EEG, resting-state activity can provide valuable predictive information regarding network activity after a stroke, but has limitations with regard to spatially localizing the sources, or regions of interest, within the network. Stroke can affect the synchrony of electrical oscillations in neural networks and these changes in network coherence can be associated with neurological deficits. In individuals with sub-acute stroke, functional connectivity of resting-state EEG correlated with motor performance. Individuals with stroke presented with disrupted alpha band connectivity where the spatial distribution of alpha activity reflected the pattern of motor and cognitive deficits of the individual participant (106). Even 1 month after stroke, measures of delta and alpha power were correlated with stroke severity scores (107). Focal brain lesions affect functional brain networks. In individuals 3 months after ischemic stroke, the synchrony of alpha band oscillations decreased between affected brain regions with the rest of the brain and this decrease was related to cognitive and motor deficits (108). Resting-state EEG can measure the synchronization of neuronal firing, and this can occur in the form of phase coupling or amplitude correlation. Behavioral performance after a stroke can be predicted by two distinct resting-state EEG coupling patterns: (1) amplitude of beta activity between homologous regions and (2) the lagged phase synchronization in EEG alpha activity from one brain region to rest of the cortex (109). A disruption of these coupling patterns is found to be associated with neurological deficits in individuals with stroke (109). Robot-aided rehabilitation programs are a relatively new and promising therapy, promoting brain plasticity and supporting improvements in upper extremity motor control. In a pilot study of seven individuals with stroke, 12 weeks of robotic rehabilitation decreased upper limb impairment and changed brain connectivity as indicated by altered coherence in the high beta band (24–33 Hz) (110). These studies demonstrate the ability of EEG to provide information about the patterns of impairment and recovery after stroke.

#### Transcranial Magnetic Stimulation

**Figure 2:**
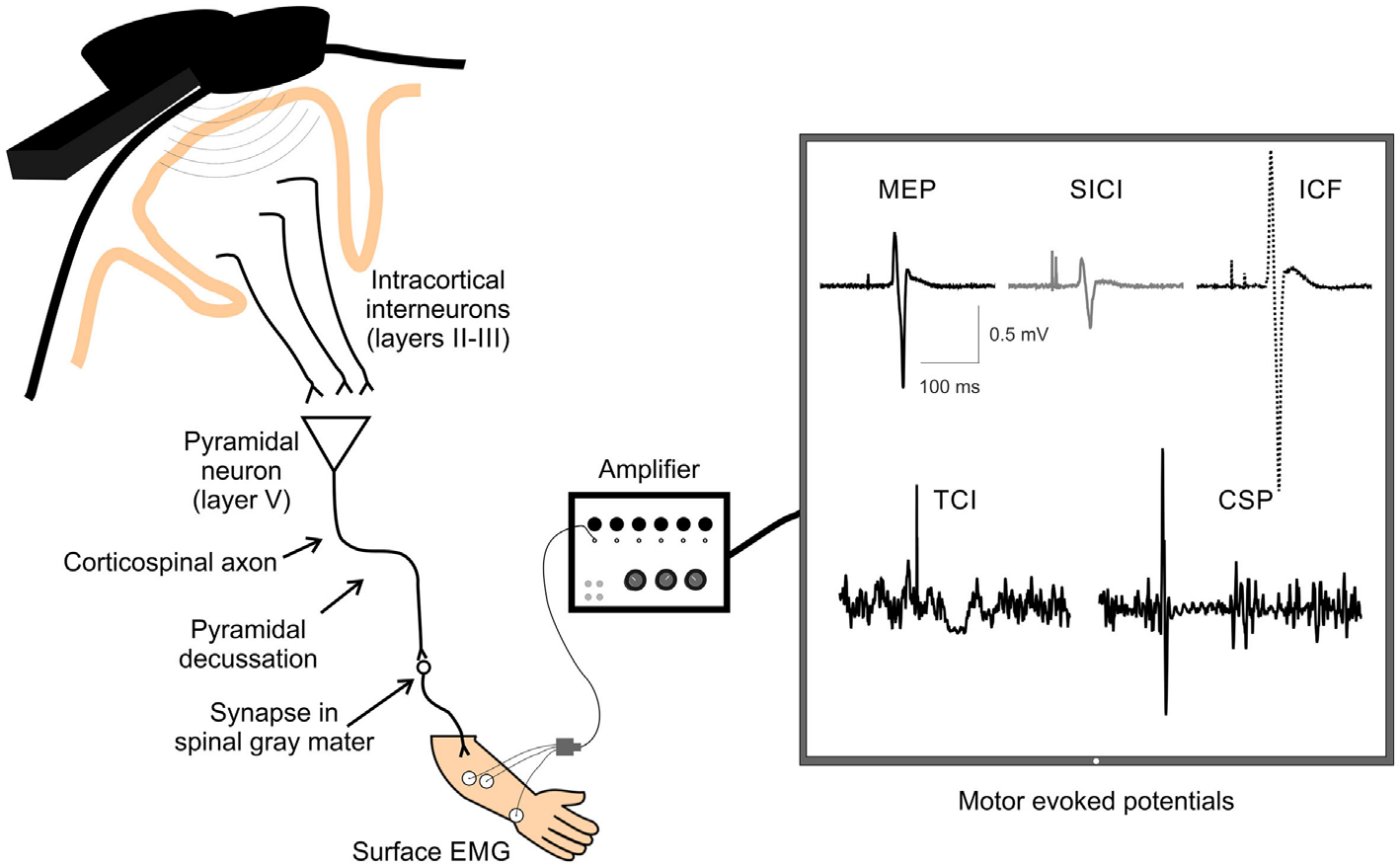
**A schematic of TMS-evoked measures of single and paired-pulse corticospinal excitability**. Examples of TMS-evoked measures of corticospinal excitability recorded by surface electrodes over the extensor carpi radialis (ECR) muscle. Displayed are examples of motor-evoked potential (MEP), short-interval intracortical inhibition (SICI), intracortical facilitation (ICF), transcallosal inhibition (TCI), and cortical silent period (CSP). TMS, transcranial magnetic stimulation; EMG, electromyography.

Transcranial magnetic stimulation is a useful way to non-invasively measure and modulate cortical excitability. TMS activates neurons in the cortex under the coil, which at high enough intensities transsynaptically depolarizes corticospinal output neurons. The corticospinal volleys activated by TMS reach the target muscle and can be recorded by surface electromyography (EMG) (111). Multiple single and paired-pulse techniques can be used to index neuroplasticity, providing useful information about how stroke and subsequent interventions modify brain function (see Figure 2 for overview).

##### Single Pulse

###### Motor Thresholds

In order to account for individual responses to TMS across individuals, a standardized motor threshold value is determined. Resting motor threshold is most commonly defined as the lowest percent of stimulator output that is required to produce a motor-evoked potential (MEP) with a peak-to-peak amplitude of 50 μV on five out of 10 trials while the individual is at rest (112). Similarly, active motor threshold is defined as the lowest percent of stimulator output that is required to produce an MEP with a peak-to-peak amplitude of 200 μV on five out of 10 trials while the individual maintains a light background contraction (113). Threshold values are often used to determine the stimulation intensity to use in the assessment and modulation of cortical excitability with TMS techniques.

###### MEP Input–Output Curves

Motor-evoked potential input–output (IO) curves utilize single-pulse TMS over a range of intensities to measure the increase in excitability within the corticospinal system in response to increased stimulus intensity, as indexed by MEP amplitude (114, 115). The linear slope of the curve (115) or area under the curve (116) produced by increasing stimulator intensity is quantified as a representation of the ability of the excitability of the M1 representation to be up-­regulated, and the strength of the corticospinal connections. MEP IO curves can be measured while the participant is at rest, or during a sustained contraction. Resting MEP IO curves activate lower threshold neurons, while active MEP IO curves utilize the voluntary contraction to activate higher threshold neurons, thus stimulating unique neuronal pools, which may have different functional significance (117).

###### M1 Cortical Mapping

Single-pulse TMS can also be utilized to probe the excitability of M1 in terms of quantifying the distribution and amplitudes of MEPs in the target muscle(s). TMS mapping of M1 follows the principles of motor homunculus (118) where stimulation of different motor regions produces systematic responses in the corresponding peripheral musculature. The amplitudes and distribution of MEPs when different scalp sites are systematically stimulated can be analyzed and displayed as topographical maps showing the greatest activity produced from a corresponding scalp location over M1. Mapping the M1 representation of particular muscles is used to understand the healthy and pathological cortex, as well as to map change in neuronal representation of muscle groups over time or following an intervention (119–129).

###### Silent Period

When single-pulse TMS is applied while holding a slight contraction in the contralateral limb, a cortical silent period (CSP) is produced, which presents a prolonged reduction in EMG activity following the MEP (130–132). The CSP originates largely from activation of inhibitory cortical and spinal interneurons, and there is evidence that the latter half of the CSP is associated with GABA_B_-like activity at the cortical level (133). Therefore, single-pulse TMS can be indicative not only of motor cortical excitability, or increases in corticospinal tract excitability in response to increasing stimulator output, but also inhibitory circuit activity within the corticospinal system.

Transcallosal inhibition (TCI), important in interhemispheric communication, can be quantified via an ipsilateral silent period (iSP) derived from single-pulse TMS (134, 135). Specifically, during a sustained unilateral muscle contraction, a single TMS pulse over the ipsilateral M1 is delivered to evoke a reduction in the background EMG activity in the ipsilateral muscle, known as the iSP. Since the iSP is diminished or absent in patients with lesions of the corpus callosum (135, 136), it is likely a result of inhibition via transcallosal projections.

##### Paired Pulse

###### Intracortical Inhibition and Facilitation

The excitation of M1 pyramidal neurons that ultimately translates into corticospinal output to target muscles is also influenced by intracortical circuitry within the motor cortex. Inhibitory intracortical circuitry within M1 influences corticospinal output, and can be quantified using TMS. Specifically, short-interval intracortical inhibition (SICI) and long-interval intracortical inhibition (LICI), quantify inhibitory circuitry. SICI is produced when two TMS pulses (a subthreshold conditioning stimulus followed by a suprathreshold test stimulus) are administered over M1 with an interstimulus interval (ISI) of 1–6 ms and results in a decreased MEP amplitude than that elicited by a single TMS pulse alone (137). Intracortical facilitation (ICF), from 10 to 15 ms after the stimulation, measures the facilitatory circuits in M1. The protocol for measuring ICF is identical to that with SICI (subthreshold conditioning stimulus and suprathreshold test stimulus), with only the ISI differing. At longer ISIs of 50–200 ms, there is again a period were inhibition is produced due to paired-pulse TMS called LICI (138, 139). Unlike SICI, LICI is evoked with two identical suprathreshold pulses. SICI is likely mediated by GABA-A (140) and LICI by GABA-B (133, 141–143) receptor-mediated circuitry, due to the differences in the time course of activation of the respective circuitry (Figure 1, Section IIF). ICF appears to be mediated by different neural circuitry than SICI (144), and glutamate may play a role in mediating ICF (145). Assessing these inhibitory and facilitatory circuits is an important component of understanding how neuroplastic change may be mediated and underlies associated behavioral changes, functional improvement, and assessment of neurological injury (i.e., stroke).

###### Short-Afferent Inhibition and Long-Afferent Inhibition

Measures of short (SAI) and long-afferent inhibition (LAI) use single-pulse TMS in conjunction with peripheral nerve stimulation to examine the integration of sensory information into the motor output system. Specifically, an electrical stimulation is delivered at the contralateral median nerve prior to a TMS pulse delivered over M1 while the participant is at rest, which results in a reduced MEP relative to a single pulse alone. SAI applies this technique with an ISI of 20 ms and LAI utilizes an ISI of 200 ms (130, 146–150). SAI provides only enough time for activation of the primary somatosensory cortex and secondary somatosensory cortex, whereas LAI is long enough to ensure activation of primary somatosensory cortex, bilateral secondary somatosensory cortex, and contralateral posterior parietal cortex (130). While the mechanisms underlying both SAI and LAI have not been described, they provide information on the impact of peripheral nerve stimulation on M1 excitability, which is an important component to consider when studying sensorimotor integration in regards to neuroplasticity and neurological injury.

###### TMS Assessment of Cortical Excitability and Connectivity in Stroke

Several methods of TMS assessment have shown that there is altered brain excitability and connectivity during all phases post-stroke (acute, sub-acute, and chronic). In approximately the first week after stroke, the ability to elicit MEPs in the paretic limb after single-pulse stimulation over the ipsilesional hemisphere predicts good recovery (151–156). A lack of elicited MEPs in the paretic limb along with increased MEP amplitudes in the non-paretic limb after contralesional stimulation predicts poor motor recovery (31, 157), although this is not always the case (151, 158, 159). The appearance of MEPs where there were none before and improvement of TMS measures of corticospinal integrity during the first few months of recovery (160–162), both correlate with better functional outcome. An imbalance of motor cortex excitability (decrease lesioned cortex excitability and overly increased excitability of contralesional cortex) occurs following severe stroke and a restoration of balance is associated with functional recovery (151, 157, 162, 163). Several studies utilizing motor cortical mapping have show that there are a decreased number of excitable scalp sites over the ipsilesional compared to contralesional cortex (160, 164–168), which has been suggested to indicate a hemispheric imbalance between the cortices that accompanies motor impairment of the more affected limb.

After stroke, measures of intracortical inhibition and excitation within the ipsilesional hemisphere are altered. There is increased inhibition as measured by a prolonged CSP after subcortical stroke (169). Conversely, SICI and LICI are suppressed (158, 170, 171), and ICF remains within normal ranges (172–174). Recent reports have shown that SAI is reduced in the acute phase of stroke, where increased suppression of SAI has been correlated with better motor function 6 months after stroke (175). In the contralesional hemisphere, motor thresholds and MEP amplitudes remain generally normal (151, 162, 173, 176–181), but SICI is suppressed in some (158, 172, 173, 177).

The connectivity between hemispheres is also altered following stroke, showing asymmetric transcallosal interactions. Several studies show that ipsilesional M1 generates less TCI than usual (177, 182), and contralesional M1 continues to demonstrate normal, or even increased, levels of interhemispheric inhibition (IHI) (183, 184). The net result is increased inhibition acting on ipsilesional M1 (183) that can depress ipsilesional M1 excitability. These changes may interfere with neuroplasticity in ipsilesional cortex (4, 185, 186), as increased IHI from contralesional M1 onto ipsilesional M1 reduces excitability in neurons that survived the stroke (177, 187) and is associated with more severe functional deficits (183, 184). Additionally, work from our group with chronic stroke participants has found increased TCI from the ipsilesional to contralesional M1 while maintaining a contraction, suggesting greater inhibitory signals sent from the ipsilesional to contralesional M1 (188). Further, we have recently shown that contralesional TCI was negatively correlated with hemiparetic arm function and impairment, demonstrating decreased inhibition from the contralesional to ipsilesional hemisphere is associated with greater impairment (189). Therefore, bilateral alterations in cortical excitability and circuitry are associated with the degree of motor impairment and post-stroke recovery.

#### Modulation of Cortical Excitability with Repetitive TMS

Repetitive TMS can be applied in specific patterns to uniquely modulate cortical excitability; the effects of rTMS may last for periods of time exceeding that of stimulus application, from minutes to an hour beyond stimulation (190–192). Therefore, rTMS can be used to index neuroplasticity or enhance cortical excitability before a behavioral intervention, such as skilled motor practice (193, 194).

Repetitive TMS, when applied in specific patterns, can excite or inhibit a local cortical region for a short duration. rTMS can be applied at low frequencies of under 1 Hz that suppresses excitability in the targeted area, or at high frequencies over 1 Hz, which transiently excites the targeted area for ~15 min (195). Similarly, theta burst stimulation (TBS) uses a 5-Hz stimulation pattern, with triplets of 20 Hz stimulation, to inhibit or facilitate cortical excitability if the TBS is applied continuously (inhibitory cTBS), or intermittently (facilitatory iTBS), respectively (190). The effects of cTBS and iTBS can last up to 60 min post-stimulation (190, 191). Importantly, the specific effects of cTBS and iTBS show substantial inter-individual variability, which likely depends upon which interneuron populations are activated by the TMS pulse (196). rTMS protocols, like TBS, have been shown to modulate cortical excitability, and at times behavior, when applied over motor-related areas, such as M1 (190), contralateral M1 (197, 198), the SMA (199), the dorsal premotor cortex (PMd) (200), the primary somatosensory cortex (S1) (194), area 5 (201), as well as non-motor areas, such as the cerebellum (202) and the DLPFC (203). Not only does rTMS modulate cortical activity directly below the magnetic coil, but activity in remote cortical and subcortical regions can be modified by application of rTMS over a single cortical target (204). Specifically, changes in MRI activity can be detected in M1/S1, SMA, PMd, cingulate motor area, the putamen, and thalamus after rTMS over left hemisphere M1 or S1 (204). These methods for modulating cortical excitability are thought to mimic early stages of long-term potentiation (LTP) or long-term depression (LTD)-like mechanisms, and are proposed to be dependent upon NMDA receptors (205). Due to the ability to modulate cortical excitability in motor and non-motor-related cortical areas beyond the time of stimulation itself (206), rTMS has been utilized by researchers to developed protocols to test whether the application of stimulation alone, or in conjunction with other behavior and therapy can further rehabilitation from neurological impairment, such as stroke.

##### Repetitive Brain Stimulation as an Intervention After Stroke

Since rTMS is known to modulate cortical excitability in local and remote regions to the areas stimulated, it has been suggested to be a viable therapeutic approach to aid in the recovery of motor function after stroke (207), yet there is accumulating evidence that the response to rTMS is inconsistent and variable (34, 193, 194). When targeting stimulation over M1, rTMS has been delivered in isolation (34, 208–210) and in combination with rehabilitation training (193, 194, 211, 212) in individuals with stroke. Since the effects of rTMS can outlast the period of stimulation itself (190, 206), the prevailing thought is that the aftereffects may be capitalized on by pairing it with skilled motor practice and/or rehabilitation training to promote neuroplastic change (193, 194, 213).

**Figure 3:**
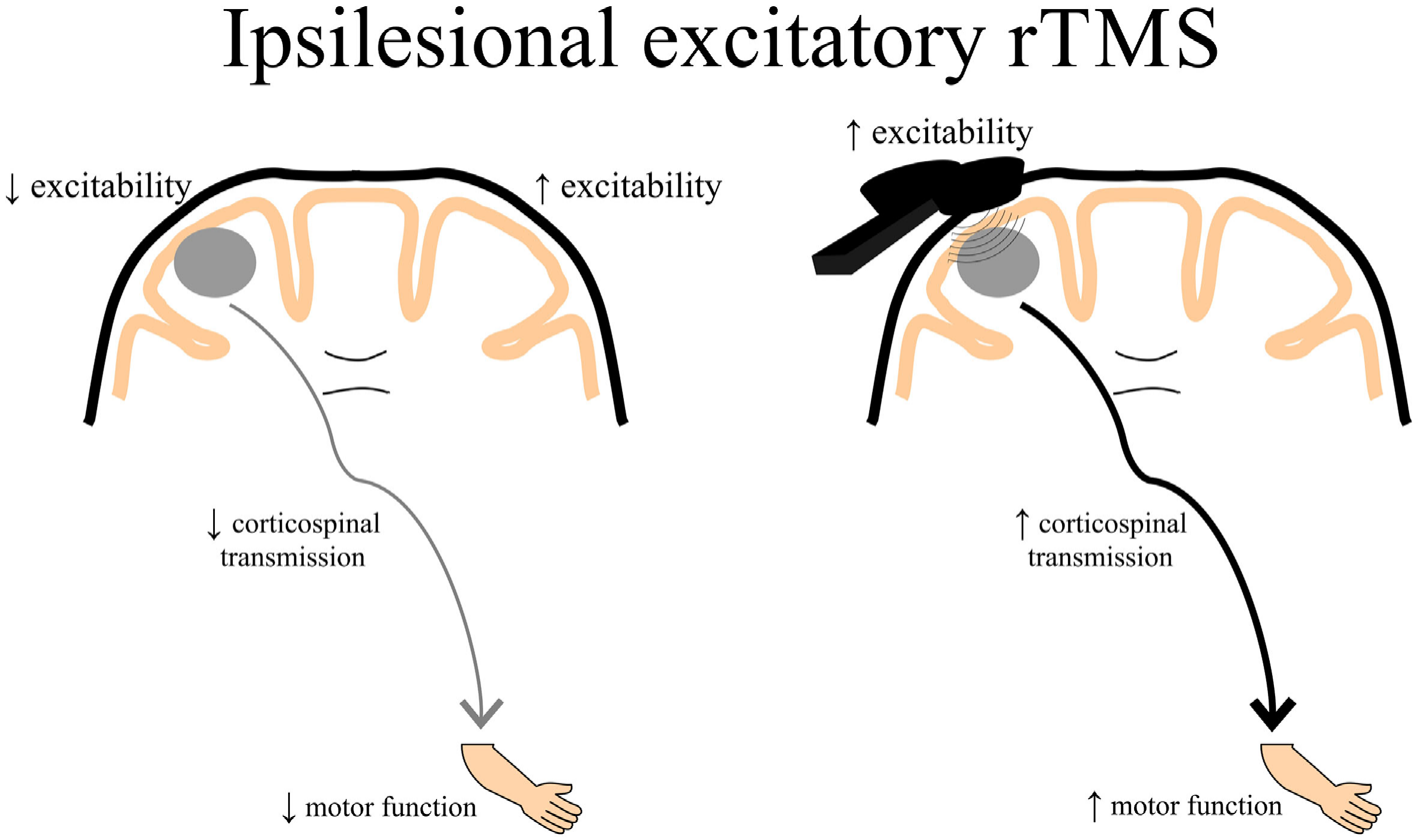
**A schematic of the theoretical effects of excitatory rTMS over the ipsilesional cortex**. Decreased ipsilesional cortical excitability may contribute to decreased corticospinal transmission resulting in diminished motor function of the paretic upper limb. Ipsilesional excitatory rTMS may increase the excitability of the damaged cortex, thereby contributing to enhanced corticospinal transmission potentially leading to better motor function of the paretic upper limb. rTMS, repetitive transcranial magnetic stimulation.

**Figure 4:**
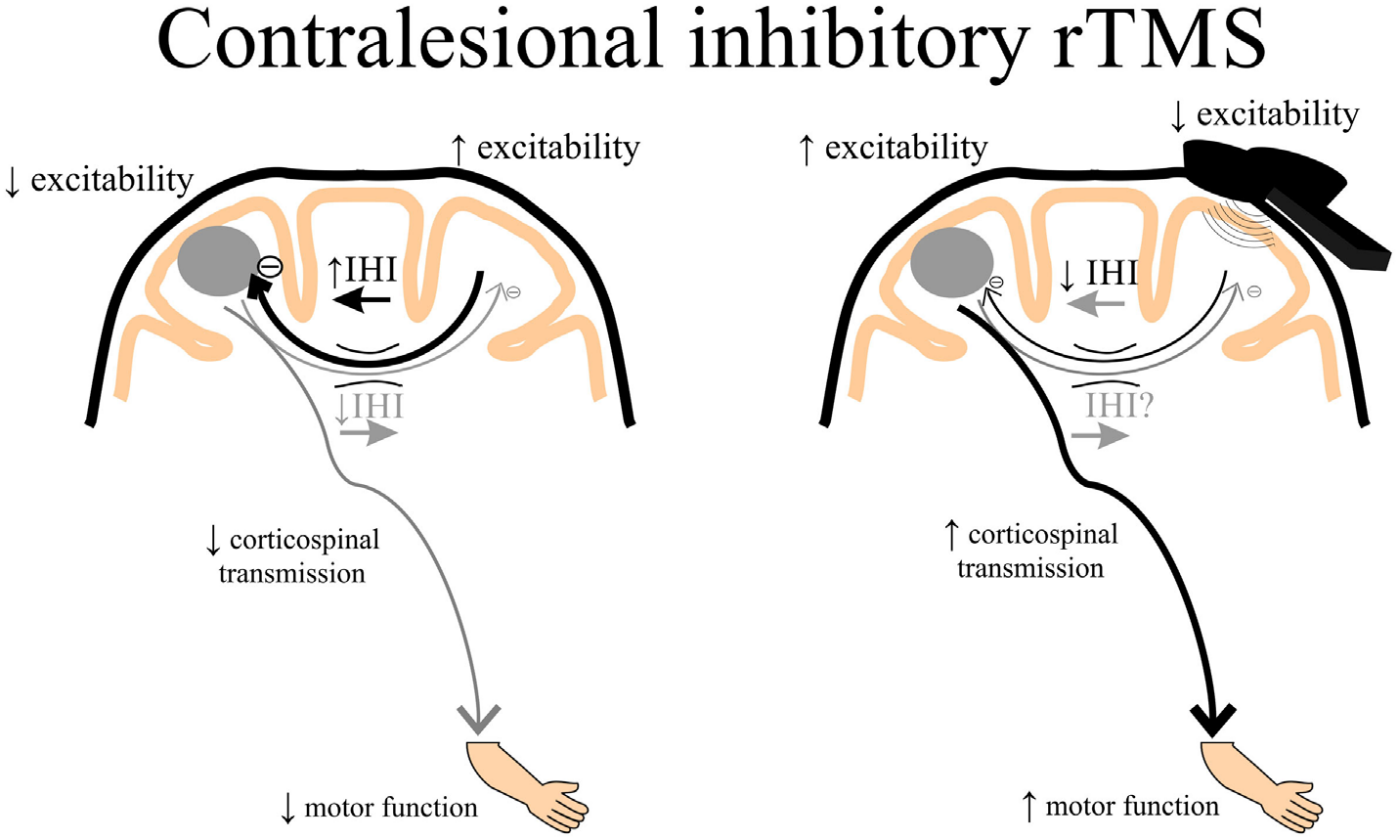
**A schematic of the theoretical effects of inhibitory rTMS over the contralesional cortex**. Increased interhemispheric inhibition (IHI) from the contralesional to ipsilesional cortex via the corpus callosum may contribute to decreased ipsilesional corticospinal excitability and diminished motor function of the paretic upper limb. Contralesional inhibitory rTMS may suppress contralesional to ipsilesional IHI and assist in improving ipsilesional corticospinal transmission, potentially leading to better motor function of the paretic upper limb. rTMS, repetitive transcranial magnetic stimulation.

Theoretically, rTMS can be used to increase cortical excitability in the ipsilesional cortex by directly applying excitatory rTMS over the ipsilesional hemisphere (Figure 3) or by applying inhibitory rTMS over the contralesional to potentially decrease abnormally increased inhibition to the lesioned M1 (Figure 4). This manipulation of cortical excitability is supported by observations of imbalanced IHI after stroke (214). Impaired motor performance following stroke is often attributed to a disruption in IHI where an overactive contralesional area suppresses the activity of the lesioned hemisphere.

##### Repetitive Brain Stimulation as an Intervention After Stroke: Ipsilesional Stimulation

Studies have shown promising preliminary findings using high-frequency excitatory (>1 Hz) rTMS applied over the ipsilesional hemisphere. One study showed that 3-Hz rTMS over the ipsilesional hemisphere for 10 days combined with passive limb manipulation, which gradually increased to active manipulation of the paretic limb, resulted in improvements in function and recovery of MEPs in certain individuals, with no relationships between improvements in function and MEP increases (215). Another study demonstrated increases in MEPs and improvements in a sequential finger motor task when 10-Hz rTMS was applied over the ipsilesional M1, and that cortical excitability was associated with improvements in motor learning (216). Similarly, improvements in motor skill learning have been shown when 5-Hz rTMS is applied over ipsilesional S1 (193) and this improvement is dependent on the white matter volume in the somatosensory cortex in the lesioned hemisphere (24). Although variable depending on stroke location, individuals with subcortical stroke only showed improved movement kinematics after 10-Hz rTMS over ipsilesional M1, whereas hand dexterity actually deteriorated in the majority of those with cortical stroke (217). This study also found that rTMS reduced activation of contralesional cortex for those with subcortical stroke, and caused bilateral activation of primary motor and sensory areas in those with cortical stroke (217). The authors concluded that it is likely that the extent and location of stroke may determine the beneficial response to ipsilesional excitatory rTMS. Studies have also reported little effects of applying excitatory rTMS over the ipsilesional cortex. Talelli and colleagues used iTBS over ipsilesional M1 followed by intensive physiotherapy of the paretic upper limb for 10 days that did not show any significant improvements (212). Another study combined 20-Hz rTMS over ipsilesional M1 with CIMT in chronic stroke for 2 weeks, finding that no additional improvements beyond that of CIMT alone were observed except slightly lower motor thresholds (218). However, it could be that the pairing of excitatory rTMS over the index finger muscle representation in M1 followed by reaching, grasping and other gross arm movements contributed the lack of positive effects in the above two studies. A recent study suggested that 10-Hz rTMS applied over ipsilesional M1 delivered 5 days per week for 2 weeks enhanced motor function of the paretic limb only in those with subcortical stroke and those who presented with MEPs immediately after the intervention and at a 2-week follow-up (219).

##### Repetitive Brain Stimulation as an Intervention After Stroke: Contralesional Stimulation

An alternative to directly enhance ipsilesional M1 excitability by applying excitatory rTMS over the lesioned hemisphere is to deliver inhibitory rTMS over contralesional M1. This approach potentially releases contralesional IHI and indirectly enhances ipsilesional M1 excitability. Some studies demonstrate that low-frequency inhibitory (<1 Hz) rTMS or cTBS applied over the contralesional hemisphere improves hand function (209, 210), reach-to-grasp movements (220), motor learning (194), and brief improvements in hand dexterity, which was associated with a reduction in TCI to the ipsilesional M1 (221).

Other studies have investigated functional brain activation changes following rTMS in stroke. One study showed a significant increase in the peri-infarct fMRI-related activity in the ipsilesional M1 after 6-Hz low-frequency rTMS over the contralesional M1 (208). Another study demonstrated improved motor performance of the paretic hand following 1-Hz rTMS over the contralesional M1 that was associated with a decrease in over-activation of contralesional M1 activation during paretic hand movements (222). Additionally, connectivity between SMA and M1 within the ipsilesional hemisphere was enhanced after inhibitory rTMS over contralesional M1 (222). Recently, it was shown that 10 sessions of 1-Hz rTMS over contralesional M1 showed a change in contralesional plasticity (35), and that mild improvements in motor ability was associated with more normal transcallosal white matter. These data suggest that the condition of callosal white matter may influence the impact of contralesional rTMS on recovery of motor function after stroke (35).

##### Variability in Response and Application of rTMS in Stroke

Overall, the reported effects of rTMS in individuals with stroke are moderate (223) but inconsistent (24, 34, 193, 194, 224). Varied effects are noted regardless of what type of rTMS is employed (34, 193, 194) and irrespective of the targeted brain region (34, 193, 194, 216, 225). Further, research to date has suggested that varied responses to rTMS are not explained by simple demographic factors, such as age, sex, or stroke severity (24, 34). There are several potential reasons for this variability, such as stroke location and extent (217, 226), post-stroke duration (219, 225), presence of MEPs (31, 219, 227), hemispheric dominance pre-stroke (228), callosal (33, 189, 229) and corticospinal structural integrity (24, 34, 189), cortical target location for rTMS (193, 219, 224), brain-derived neurotrophic factor genotype (230), different interneuron populations activated by TMS (196), and combination with a well-controlled motor learning task or individualized physical therapy. Despite its broad use, a comprehensive understanding of the physiologic effects of rTMS on the brain is lacking. Further, there is no consensus on which brain region to stimulate, whether it is somatosensory (193) or motor execution (194, 216) or preparation (231) areas, and if stimulation should be applied over the ipsilesional or contralesional hemisphere (194).

Although rTMS demonstrates great potential to enhance post-stroke recovery future work is needed to address the issue of response variability. With a greater understanding of the factors driving response variability, we will be better able to target rehabilitation to the individual.

## Multimodal Assessments

### Multimodal Neuroimaging: Combined TMS, MRI, and EEG Assessment After Stroke

Few studies have utilized multiple methods of neuroimaging in order to predict motor function and impairment due to stroke (188, 189, 229, 232, 233). Studies have shown that those with decreased MEP amplitudes also have a weaker paretic hand, with greater activation in the ipsilesional M1 as recorded by task-based fMRI (234). A study using TMS to assess corticospinal integrity via single-pulse assessment of MEPs and fMRI during isometric hand gripping determined that in patients with less corticospinal excitability, there was an associated increase in activation of contralesional premotor and cerebellar areas (232). The suggestion from these combined methods is that other non-primary motor cortical areas may be playing a functionally relevant role in controlling force production in more severely affected individuals with stroke. A combined paired-pulse dual coil TMS and fMRI study showed that both TMS and fMRI neurophysiological function in the contralesional PMd was associated with the degree of impairment (233). Specifically, a lack of inhibition from contralesional PMd to ipsilesional M1 measured by paired-pulse TMS and greater activation of PMd during hand-grip was correlated with the level of clinical impairment. The authors suggested that contralesional PMd may support recovery in ipsilesional M1 (233).

Stinear and colleagues have demonstrated through DTI and TMS that weak or absent MEPs evoked ipsilesionally and greater asymmetry in FA of the posterior limb of the internal capsule are predictive of poor motor recovery (227). Recent work from our lab has demonstrated the utility of combined TMS and MRI measures to predict motor function (188). Specifically, bilateral hand dexterity was found to correlate with resting motor threshold and precentral gyral thickness. Those with higher resting motor thresholds and decreased precentral gyral thickness presented with decreased bilateral hand dexterity. Furthermore, increased levels of TCI were associated with greater midcallosal white matter volume (188). In another study, we demonstrated that altered microstructure of transcallosal fiber tracts in anterior sub-regions were associated with TCI and upper extremity impairment in chronic stroke (189). Specifically, anterior transcallosal tract FA and TCI from the non-lesioned to lesioned M1 predicted a unique amount of variance in upper limb impairment. Those with less FA in anterior sub-regions of the corpus callosum and less TCI were those presenting with greater upper limb impairment (189). Another recent study combined fMRI, DTI, and TMS in the assessment of hemispheric balance between ipsilesional and contralesional cortices (229). Task-based fMRI lateralization to the ipsilesional hemisphere was associated with better TCI and stronger ipsilesional motor-related area output via DWI tractography. These studies demonstrate the usefulness of combining multiple methods of neuroimaging along with measures of TMS in order to more comprehensively assess and predict motor function and impairment. Utilizing multimodal neuroimaging can be used in future investigations to aid in identifying optimal biomarkers of stroke recovery and to predict response to rehabilitation in order to maximize treatment outcomes.

A novel multimodel neuroimaging approach combines TMS with EEG. TMS and EEG may be used in combination in real-time in order to directly characterize local and distributed cortical activity, providing a rich source of temporally specific data to determine causal mechanisms of cortical responses to TMS in humans *in vivo* (235–238). Another advantage of this approach is the ability to stimulate any cortical regions and record the evoked activity using EEG, subverting the need to record peripheral responses via surface EMG, which can prove difficult in the ipsilesional hemisphere. Although this has not been utilized in stroke, the combined technique of TMS-EEG may provide new insights into cortico-cortical connectivity in sensorimotor recovery after stroke due to spontaneous recovery and with interventions (behavioral, stimulation, pharmacological, etc.) not able to be captured before.

### Multimodal Neuroimaging: TMS and MRI Assessment of rTMS-Based Interventions After Stroke

Very few studies have utilized multimodal neuroimaging with TMS to identify the underlying neurobiology of sensorimotor recovery from stroke (31, 188, 189, 227, 229, 233), and research is scarce in the investigation of an intervention using multimodal imaging with rTMS (24, 35). These studies have demonstrated the usefulness of combining imaging of cortical and subcortical structures with neurophysiological data acquired from TMS in order to better predict aspects of upper limb motor recovery and the potential response to rTMS (24, 34, 35). Carey et al. demonstrated that those with greater structural integrity of the posterior limb internal capsule of the ipsilesional hemisphere demonstrate greater response to contralesional rTMS and the behavioral improvements associated with rTMS (34). Transcallosal FA was shown to correlate with the degree of behavioral improvements due to contralesional rTMS, indicating the DTI-derived measures may aid in individually tailored interventions when considering using contralesional rTMS to potentially induce transcallosal neuroplasticity (35). Recently, we have shown that increased ipsilesional S1 white matter volume was associated with the degree of skill learning improvement when 5-Hz rTMS was applied over S1 before motor skill practice (24). These studies suggest that data acquired from structural and functional imaging may be used to categorize those who respond to rTMS in order to personalize application in a rehabilitation setting.

## The Future of Multimodal Neuroimaging for Personalized Therapy

Recently, there have been several models proposed to categorize individuals for personalized treatment based on multi-neuroimaging methods (227, 239). The “predicting recovery potential (PREP) algorithm” has been introduced and suggested that patients who present with an ipsilesional MEP have the best prognosis for recovery, and intensive unilateral therapy of the paretic limb is recommended. However, those who do not present with an ipsilesional MEP are divided into two categories: (1) low asymmetry in FA of the corticospinal tract (greater integrity of the ipsilesional corticospinal tract), with a prognosis of limited functional improvement and (2) high asymmetry in FA of the corticospinal tract (less integrity of the ipsilesional corticospinal tract) with the poorest prognosis for functional improvement. Those with low corticospinal tract asymmetry are recommended to receive “primed” ipsilesional brain stimulation and augmented training of the paretic upper limb. However, if there is an absence of an MEP after stimulating the ipsilesional M1 with a relatively high hemispheric asymmetry of FA, the recommendation of therapeutic intervention is modified to include stimulation of the contralesional M1 along with augmented bilateral therapy to engage the contralesional and ipsilesional cortices (31, 227). Di Pino and colleagues (239) similarly have suggested a bimodal balance-recovery model that proposes a personalized application of rTMS (or other types of non-invasive brain stimulation) depending on structural reserve of the central nervous system, along with clinical and neurophysiological data from multiple imaging sources. This bimodal balance-recovery model attempts to account for the possibility of interhemispheric competition and the fact that the contralesional hemisphere may serve to support recovery of function after stroke (239).

These studies suggest that a combination of neuroimaging methods will likely benefit in the assessment of stroke-related damage and personalized treatment strategies, particularly when using rTMS (or other types of non-invasive brain stimulation) for individuals following stroke. However, there will always be a risk of mislabeling participants, resulting in a substandard care. For this reason, we must continue to utilize new technologies to broaden our understanding of stroke recovery, improving both diagnostic abilities and interventions. For instance, in an individual who does not present with an ipsilesional MEP perhaps simultaneous TMS-EEG could be used to test if cortical activity is evoked by ipsilesional TMS, making it possible to narrow down the site of impairment. This could be very useful information, giving a more accurate prognosis and identifying the ideal pathway to target for recovery. As advancements in neuroimaging continue to impact research in stroke recovery, personalized therapy will become more reliable and utilized, and new interventions will become possible.

## Conclusion

The information provided above strongly suggests the potential for multimodal imaging in future neuroplasticity and rehabilitation studies after stroke. Structural and functional imaging and physiological assessments have all provided important insights into both the pathology of stroke and mechanisms underlying neurological recovery. Table 1 lists the major benefits/limitations of each imaging method covered in this review. Additionally, we have also highlighted the potential of non-invasive brain stimulation as an important therapeutic approach. Although many studies have found rTMS improves recovery an increasing number are failing to find benefit. Numerous technical factors affect rTMS interventions, including the site targeted, type of stimulation, and number of stimulation sessions. However, the variability in response to rTMS also highlights the importance of understanding individual differences in response, which likely depend on a variety of biological factors, such as, age, time after stroke, lesion size, and location, which in turn impact patterns of functional and structural connectivity. Advances in neuroimaging are improving the ability to predict the patterns of structural and functional connectivity best suited to specific interventions. In the near future, novel-individualized interventions will be able to optimize recovery after stroke.

**Table 1:** **Cost/availability are ranked relative to the other imaging methods**.

Method	Benefits	Limitations	Cost/availability
Volumetric analysis	Quantification of brain volumes from basic (T1) structural scans	Limited accuracy in individuals with lesions	$$/+++
DW-MRI	Assesses microstructural characteristics of white matter	Tractography results are variable across methods and sensitive to movement	$$$/++
MRS	Quantification of neurotransmitter levels in defined area	Requires technical expertise and expensive coil for acquisition	$$$/+
fMRI	Identifies patterns of brain activation at high spatial resolution	Poor temporal resolution and is limited to participants who can complete task	$$$/++
Resting-state fMRI	Not dependent on task completion	Sensitive to movement	$$$/+
TMS	Assessment and modulation of cortical excitability and plasticity	Requires specialized equipment and trained personnel	$$/+
EEG	Provides information on functional integrity of cortex and has high temporal resolution	Poor spatial resolution and is limited to cortical activity	$/+++

*Increasing price is associated with more $ signs, and greater availability is indicated by more + signs*.

*DW-MRI, diffusion-weighted MRI; MRS, magnetic resonance spectroscopy; fMRI, functional MRI; EEG, electroencephalography; TMS, transcranial magnetic stimulation*.

## Conflict of Interest Statement

The authors declare that the research was conducted in the absence of any commercial or financial relationships that could be construed as a potential conflict of interest.
